# CircRNAs as potential biomarkers for the clinicopathology and prognosis of glioma patients: a meta-analysis

**DOI:** 10.1186/s12885-020-07446-4

**Published:** 2020-10-15

**Authors:** Xiangqian Ding, Luyao Yang, Xin Geng, Yanghong Zou, Zhigang Wang, Yao Li, Renli Qi, Wei Wang, Jinghui Li, Hualin Yu

**Affiliations:** 1 Second Department of Neurosurgery, Kunming Medical University First Affiliated Hospital, 650032, No.295, Xichang road, Kunming, Yunnan China; 2 Department of Anesthesiology, Liaocheng people’s hospital, LiaoCheng, Shandong China; 3 Graduate School of Kunming Medical University, 650500, No.1168, Kunming, Yunnan China

**Keywords:** Circular RNA, Glioma, Prognosis, Clinicopathology, Meta-analysis

## Abstract

**Background:**

An increasing number of studies have reported circular RNAs (circRNAs) as new potential biomarkers for the prognosis of gliomas. However, the overall prognostic value of circRNAs for glioma remains unclear. Therefore, this study is the first comprehensive evaluation of the clinicopathological and prognostic value of dysregulated circRNAs in the treatment of glioma patients.

**Methods:**

We systematically reviewed the online databases of PubMed, Web of Science, EMBASE, and Cochrane Library to identify studies that explored the relationship between circRNA expression and clinicopathological and prognostic factors in glioma through April 11, 2020. The quality of the included studies was evaluated by the Newcastle-Ottawa Scale (NOS) checklists. Clinicopathological features were assessed by pooled odds ratios (ORs) and 95% confidence intervals (CIs), and overall survival (OS) was assessed by hazard ratios (HRs) and 95% CIs.

**Results:**

Twenty-four eligible studies, including 22 studies of clinicopathological features, 1 diagnostic study, and 18 studies of prognosis, that included a total of 1390 patients were ultimately included in this study. Meta-analysis showed that highly expressed oncogenic circRNAs were significantly related to poor clinicopathological features (age: *P* = 0.026; tumor size: *P* ≤ 0.001; tumor grade: *P* ≤ 0.001; KPS: *P* = 0.012) and worse overall survival (OS) (HR = 2.01, 95% CI: 1.61–2.50, *P* ≤ 0.001). Moreover, we found that highly expressed tumor-suppressor circRNAs were related to better clinicopathological features (gender: *P* = 0.042; age: *P* = 0.014; tumor size: *P* = 0.022; tumor grade: *P* ≤ 0.001) and longer OS (HR = 2.70, 95% CI: 1.82–3.99, *P* ≤ 0.001).

**Conclusions:**

In conclusion, there is a significant correlation between the dysregulated expression of circRNAs and the clinicopathology and prognosis of glioma patients.

## Background

Glioma is a type of primary central nervous system tumor that originates from glial cells, accounting for approximately 80% of intracranial malignancies [1]. In recent years, although much progress has been made in the field of glioma surgery, radiotherapy, chemotherapy, and targeted therapy, the therapeutic effect is still not ideal, and the average survival time of patients is only 12–14 months [2]. With the development of high-throughput sequencing technology, research on the molecular basis of cancer has advanced, but the pathogenesis and biomarkers of gliomas are still unclear [3–5]. To improve the clinical efficacy of treatment and to understand the pathogenesis of glioma more clearly, it is urgent to conduct further research on molecular markers related to glioma.

Circular RNA (circRNA) is a type of long-chain noncoding RNA without a 5′ cap and 3′ polyadenylated tail structure that is joined by a covalent bond structure after special pre-RNAs undergo back-splicing. CircRNA is widely present in different species and a variety of human cells and has the characteristics of structural stability, high conservatism, complex regulation and tissue-specific expression [6]. Studies have shown that circRNA can act as a “molecular sponge” to adsorb microRNAs (miRNAs) to regulate gene expression, and can also play a role in directly regulating transcription and interfering with splicing mechanisms, so we believe that circRNA is closely related to the existence and development of cells like long non-coding RNAs [7–9].

Recently, the relationship between circRNAs and glioma prognosis has become the focus of research. For example, the high expression of hsa_circ_0013520 and hsa_circ_0004379 is related to poor prognosis in glioma patients and can be used as a novel prognostic biomarker for glioma [10]. However, single studies often have the disadvantages of being inaccurate and incomprehensive because of small sample sizes and the nature of being a single research project. Through searching, we did not find a meta-analysis about the prognostic value of circRNAs in gliomas. Therefore, we conducted a meta-analysis to explore the relationships of clinicopathological characteristics and prognosis with circRNA expression.

## Methods

The project design, data analysis and reporting of the results of this study were strictly implemented in accordance with the Preferred Reporting Items for Systematic Reviews and Meta-analyses (PRISMA) statement guidelines [11]. We analyzed only summary data at the research level.

### Literature retrieval strategy

We carefully reviewed the PubMed, EMBASE, Cochrane Library, and Web of Science databases for relevant articles that studied the clinical value, including the assessment of pathological features, diagnosis, and prognosis, of circRNA expression in glioma patients before April 11, 2020. We searched PubMed using the following strategy: (((“Glioma”[Mesh]) OR (((((((((((((Gliomas) OR Glial Cell Tumors) OR Glial Cell Tumor) OR Tumor, Glial Cell) OR Tumors, Glial Cell) OR Mixed Glioma) OR Glioma, Mixed) OR Gliomas, Mixed) OR Mixed Gliomas) OR Malignant Glioma) OR Glioma, Malignant) OR Gliomas, Malignant) OR Malignant Gliomas))) AND ((“RNA, Circular”[Mesh]) OR ((((((((((((circRNAs) OR Closed Circular RNA) OR Circular RNA, Closed) OR RNA, Closed Circular) OR Circular RNA) OR Circular RNAs) OR RNAs, Circular) OR circRNA) OR Circular Intronic RNA) OR Intronic RNA, Circular) OR Intronic RNA, Circular) OR ciRNA)). We also manually screened references for applicable articles.

### Eligibility criteria

The inclusion criteria were articles that (a) studied the expression level of circRNA in clinical glioma patients and reported the relationship between the expression level and clinicopathological characteristics; (b) studied the circRNA concentration of a sample and reported sufficient data on the sample size, sensitivity and specificity; (c) studied the relationship between lncRNA expression levels and overall survival (OS), and provided hazard ratio (HR) values and 95% confidence intervals (CIs), or Kaplan-Meier curves. Moreover, the exclusion criteria were (a) non-English articles; (b) studies not relevant to circRNA or glioma; (c) studies not conducted on humans; (d) studies in which the sample size was less than 30; (e) reviews, case reports, meeting records or letters; (f) studies without available data for analysis and whose authors could not be contacted.

### Quality assessment and data extraction

The quality assessment of eligible studies was independently completed by two investigators (Xiangqian Ding and Luyao Yang) in accordance with the Newcastle-Ottawa Scale (NOS). Differences were finally agreed upon through negotiation. The highest NOS score was 9, and the NOS score cut-off for high-quality studies was ≥6.

Data extraction for eligible studies was also completed independently by two investigators (Xiangqian Ding and Luyao Yang) in accordance with standardized forms. The researchers reached an agreement through consultation. Contents of the standardized form covered the following information: (a) shared information: author, publication year, study region, circRNA type, tumor type, expression level of circRNAs, and assay methods; (b) clinicopathological features: sample size, cut-off definition, gender, age, tumor size, WHO grade, tumor location, and Karnofsky performance score (KPS); (c) diagnostic information: detected sample, sample size, sensitivity, specificity, area under the curve (AUC), and 95% CI; and (d) prognostic information: sample size, cut-off definition, tumor stage, detected sample, high level, low level, survival analysis, HR, 95% CI, high vs low expression, HR availability, analysis type, follow-up month, or data extracted from Kaplan-Meier curves [12].

### Statistical analysis

We used STATA 14.0 software to perform statistical analysis of the extracted data. Clinicopathological parameters were evaluated using odds ratios (ORs) and 95% CIs, and OS was evaluated using HRs and 95% CIs. The heterogeneity of study was evaluated using chi-square test and *I*^2^ statistics. When the *I*^2^ value is > 50%, the heterogeneity was considered significant, and the analysis was performed using a random-effect model; when the *I*^2^ value was < 50%, the heterogeneity was considered nonsignificant, and the analysis was performed using a fixed-effect model [13]. When *P* was < 0.05, the difference was considered statistically significant.

To evaluate the stability and reliability of the pooled effect size, we conducted a sensitivity analysis of the included prognostic-related studies. The studies were excluded one by one and the effects were combined to observe whether the results changed significantly. If there was no major change in the results, the sensitivity is low and the results are stable and reliable; on the contrary, if the difference between the merged results after the exclusion and the original results is large, the sensitivity is high and the stability of the results is low. The publication bias of the included studies was qualitatively tested by a funnel chart and quantitatively tested by Begg’s and Egger’s tests.

## Results

### Literature search results

**Fig. 1:**
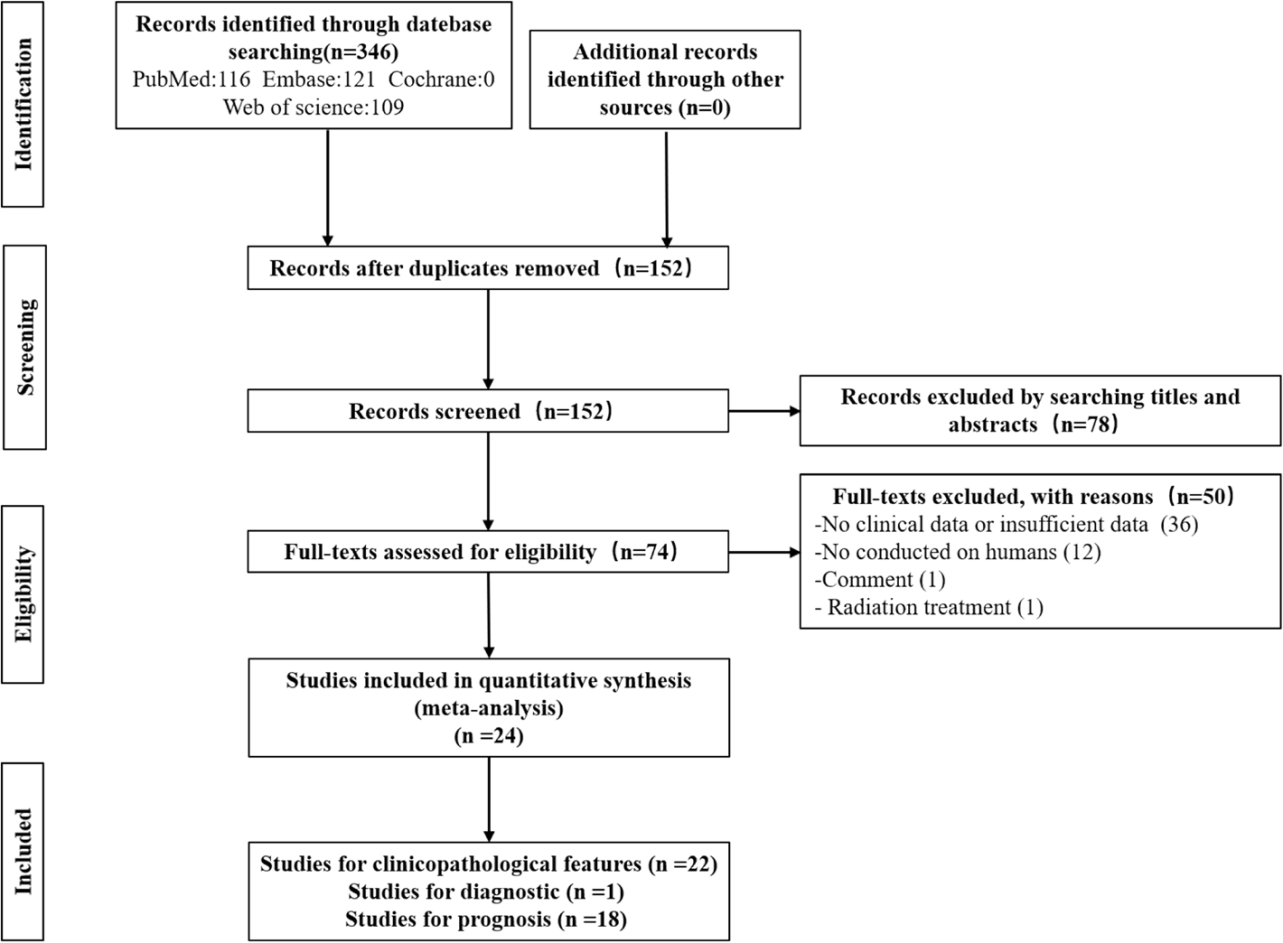
Flowchart of the study selection process

Figure 1 shows the literature retrieval process and related results. We retrieved a total of 346 related studies, including 116 from PubMed, 121 from Embase, and 109 from Web of Science. We first excluded 194 duplicate studies and then excluded 78 unrelated studies after reading the titles and abstracts. Subsequently, after reviewing the full-text of 74 studies, 50 irrelevant studies were excluded, including 36 studies without clinical data or insufficient data, 12 nonhuman studies, 1 comment, and 1 radiotherapy-related study. In summary, 24 eligible studies, including 22 studies of clinicopathological features [10, 14–34], 1 study of diagnostics [35], and 18 studies of prognosis [10, 14–28, 35, 36], including a total of 1390 glioma patients were ultimately included in this study.

### Study characteristics

Table 1 and Table 2 show the features of eligible studies related to clinicopathology and prognosis, respectively. All the samples from the 24 studies were pathologically confirmed as gliomas, and all came from hospitals in China. The publication years ranged from 2017 to 2020. The minimum sample size was 31 cases, and the maximum sample size was 99 cases. In addition, the expression levels of all circRNAs were measured using quantitative real-time polymerase chain reaction (qRT-PCR). The upregulation of 21 circRNAs was considered to have tumor-promoting effects, and the downregulation of 4 circRNAs was considered to have tumor-suppressing effects. The follow-up time ranged from 24 months to 80 months. Only one study reported the diagnostic sensitivity, specificity, and AUC data, so we did not perform a meta-analysis related to the diagnosis (Table S1). The included studies were all high-quality studies with NOS scores ≥6 (Table S2).

**Table 1:** The *p* values summary of the association between circRNAs and clinicopathological features

Author (ref.) year	Country	CircRNA	Tumor type	Expression	Assay methods	Case	Cut-off value	Gender	Age	Tumor size (cm)	WHO grade	Tumor location	KPS	Family history
Zhu [14] 2017	China	circBRAF	glioma	down-regulation	qRT-PCR	68	NA	0.331	0.011	1(5)	< 0.001	NA	0.429	NA
Wang [15] 2018	China	circ-0001649	glioma	down-regulation	qRT-PCR	64	NA	0.196	0.803	0.002(3)	0.023	0.297	NA	0.474
Qu [16] 2019	China	circ-0079593	glioma	up-regulation	qRT-PCR	60	mean	0.789	0.17	0.009(3)	0.002	0.604	NA	0.506
Lv [17] 2020	China	circ-EPB41L5	GBM	down-regulation	qRT-PCR	45	median	0.449	0.026	0.42(10cm^3^)	NA	NA	0.279	NA
Duan [18] 2018	China	circ-0074362	glioma	up-regulation	qRT-PCR	62	NA	0.806	0.074	0.04(4)	0.005	NA	NA	NA
Zhang [19] 2019	China	circ-0029426	glioma	up-regulation	qRT-PCR	59	mean	0.601	0.415	0.033(3)	0.036	0.105	NA	0.741
Yang [20] 2019	China	circ-POSTN	glioma	up-regulation	qRT-PCR	58	mean	0.787	0.77	0.033(3)	0.034	0.793	NA	0.505
Yang [21] 2019	China	circ-0034642	glioma	up-regulation	qRT-PCR	68	mean	0.312	0.201	0.014(3)	0.003	0.219	NA	0.356
Wang [22] 2019	China	circ-0005198	glioma	up-regulation	qRT-PCR	44	fold change	0.51	0.698	0.014(3)	0.012	0.543	NA	1
Meng [23] 2019	China	circ-SCAF11	glioma	up-regulation	qRT-PCR	40	NA	0.612	0.153	0.015(3)	0.178	NA	0.317	NA
Lyu [10] 2020	China	circ-0013520	glioma	up-regulation	qRT-PCR	92	mean	0.747	0.511	< 0.001(3)	0.004	NA	0.008	NA
Lyu [10] 2020	China	circ-0004379	glioma	up-regulation	qRT-PCR	92	mean	0.799	0.641	< 0.001(3)	0.005	NA	0.006	NA
Lu [24] 2019	China	circ-0001730	glioma	up-regulation	qRT-PCR	99	NA	0.132	0.611	NA	0.004	NA	NA	NA
Liu [25] 2020	China	circ-CDC45	glioma	up-regulation	qRT-PCR	50	median	0.561	0.321	0.016(3)	0.01	0.571	NA	0.496
Ding [26] 2019	China	circ-NFIX	glioma	up-regulation	qRT-PCR	64	NA	0.4363	0.765	0.0262(3)	0.0177	NA	NA	NA
Chen [27] 2018	China	circ-0000177	glioma	up-regulation	qRT-PCR	62	NA	0.451	0.198	0.01(4)	0.02	NA	NA	NA
Chen [28] 2020	China	circ-0074026	glioma	up-regulation	qRT-PCR	60	mean	0.795	0.125	0.001(3)	0.017	0.288	NA	0.731
Zuo [29] 2019	China	circ-SMAD7	glioma	up-regulation	qRT-PCR	46	NA	0.309	0.978	NA	0.014	NA	0.0002	NA
Zhan [30] 2019	China	circ-PITX1	glioma	up-regulation	qRT-PCR	52	median	0.765	0.541	0.023(3)	0.025	0.406	NA	0.499
Xie [31] 2018	China	circ-0012129	glioma	up-regulation	qRT-PCR	31	NA	0.578	0.551	NA	0.014	NA	NA	NA
Qian [32] 2019	China	circ-0074027	glioma	up-regulation	qRT-PCR	50	median	0.529	0.345	0.045(3)	0.021	0.158	NA	0.479
Liu [33] 2019	China	circ-001350	glioma	up-regulation	qRT-PCR	53	mean	0.266	0.76	0.013(3)	0.029	0.588	NA	0.392
He [34] 2020	China	circ-MAPK4	glioma	up-regulation	qRT-PCR	30	NA	0.919	0.855	0.513(3)	0.044	NA	NA	NA

*qRT-PCR* quantitative reverse transcription-polymerase chain reaction, *NA* not available, *GBM* glioblastoma multiforme

**Table 2:** Main features of studies for prognosis analysis

Author (ref.) year	Country	CircRNA	Tumor type	Expression	Assay methods	Case	Cut-offvalue	Tumor stage	High level	Low level	Survival analysis	HR	95%CI	HR availability	Follow-up months
Zhu [14] 2017	China	circ-BRAF	glioma	down-regulation	qRT-PCR	68	NA	grade(I-IV)	33	35	OS	4.13	1.79–9.56	K-M curve	60
Wang [15] 2018	China	circ-0001649	glioma	down-regulation	qRT-PCR	64	NA	grade(I-IV)	32	32	OS	2.012	1.012–4	Reported	60
Qu [16] 2019	China	circ-0079593	glioma	up-regulation	qRT-PCR	60	mean	grade(I-IV)	30	30	OS	2.21	1.023–4.772	Reported	60
Lv [17] 2020	China	circ-EPB41L5	GBM	down-regulation	qRT-PCR	45	median	grade (IV)	23	22	OS	3.401	1.388–8.88	Reported	45
Duan [18] 2018	China	circ-0074362	glioma	up-regulation	qRT-PCR	62	NA	grade(I-IV)	30	32	OS	2.695	1.054–6.897	Reported	56
Zhang [19] 2019	China	circ-0029426	glioma	up-regulation	qRT-PCR	59	mean	grade(I-IV)	31	28	OS	2.007	1.006–4.002	Reported	60
Yang [20] 2019	China	circ-POSTN	glioma	up-regulation	qRT-PCR	58	mean	grade(I-IV)	29	29	OS	2.1	0.74–5.91	K-M curve	60
Yang [21] 2019	China	circ-0034642	glioma	up-regulation	qRT-PCR	68	mean	grade(I-IV)	36	32	OS	1.75	0.84–3.63	K-M curve	60
Wang [22] 2019	China	circ-0005198	glioma	up-regulation	qRT-PCR	44	fold change	grade(I-IV)	22	22	OS	2.47	0.66–9.2	K-M curve	60
Meng [23] 2019	China	circ-SCAF11	glioma	up-regulation	qRT-PCR	40	NA	grade(I-IV)	20	20	OS	1.64	0.59–4.59	K-M curve	60
Lyu [10] 2020	China	circ-0013520	glioma	up-regulation	qRT-PCR	92	mean	grade(I-IV)	40	52	OS	1.85	0.98–3.48	K-M curve	31
Lyu [10] 2020	China	circ-0004379	glioma	up-regulation	qRT-PCR	92	mean	grade(I-IV)	42	50	OS	1.65	0.87–3.11	K-M curve	30
Lu [24] 2019	China	circ-0001730	glioma	up-regulation	qRT-PCR	99	NA	grade(I-IV)	49	50	OS	1.55	0.29–8.11	K-M curve	24
Liu [25] 2020	China	circ-CDC45	glioma	up-regulation	qRT-PCR	50	median	grade(I-IV)	25	25	OS	1.78	0.9–3.55	K-M curve	60
Ding [26] 2019	China	circ-NFIX	glioma	up-regulation	qRT-PCR	64	NA	grade(I-IV)	36	28	OS	0.87	0.17–4.46	K-M curve	60
Chen [27] 2018	China	circ-0000177	glioma	up-regulation	qRT-PCR	62	NA	grade(I-IV)	26	36	OS	2.05	0.52–8.1	K-M curve	60
Chen [28] 2020	China	circ-0074026	glioma	up-regulation	qRT-PCR	60	mean	grade(I-IV)	30	30	OS	2.6	1.37–4.94	K-M curve	60
Li [35] 2018	China	circ-ITCH	glioma	down-regulation	qRT-PCR	60	NA	grade(I-IV)	31	29	OS	2.326	1.204–5.431	Reported	80
Peng [36] 2019	China	circ-CPA4	glioma	up-regulation	qRT-PCR	73	average	grade(I-IV)	35	38	OS	3.98	1.23–12.94	K-M curve	42

*HR* hazard ratio, *CI* Confidence interval, *qRT-PCR* quantitative reverse transcription-polymerase chain reaction, *NA* not available, *OS* overall survival, *K-M* Kaplan-Meier

### Clinicopathological features

Twenty-three circRNAs were reported in the 22 included studies related to clinicopathological features. Upregulation of 20 circRNAs was considered to have tumor-promoting effects, and downregulation of 3 circRNAs was considered to be have tumor-suppressing effects. Table 3 shows the meta-analysis results of circRNAs and clinical features of glioma patients. High expression of oncogenic circRNAs was significantly related to poor clinicopathological features (age: OR = 1.32, 95% CI: 1.03–1.68; tumor size: OR = 3.48, 95% CI: 2.61–4.63; tumor grade: OR = 4.24, 95% CI: 3.26–5.52; KPS:OR = 0.21, 95% CI: 0.06–0.71). In addition, high expression of tumor-suppressor circRNAs was significantly related to better clinicopathological features (gender: OR = 1.9, 95% CI: 1.02–3.51; age: OR = 0.46, 95% CI: 0.15–1.39; tumor size: OR = 0.49, 95% CI: 0.26–0.9; tumor grade: OR = 0.12, 95% CI: 0.05–0.27), and other clinicopathological features (tumor location, KPS, and family history of cancer) had no significant relationships.

**Table 3:** Meta-analysis results for circRNAs expression with clinicopathological features

	Tumor promoter		Tumor suppressor	
	OR (95% CI)	P value	OR (95% CI)	***P*** value
Gender (M/W)	1.25 (0.99–1.58)	0.061	**1.9 (1.02–3.51)**	**0.042**
Age	**1.32 (1.03–1.68)**	**0.026**	**0.46 (0.15–1.39)**	**0.014**
Tumor size	**3.48 (2.61–4.63)**	**0.000**	**0.49 (0.26–0.9)**	**0.022**
Tumor grade (III + IV/I + II)	**4.24 (3.26–5.52)**	**0.000**	**0.12(0.05–0.27)**	**0.000**
Tumor location (Supra/Infra)	1.22 (0.87–1.70)	0.248	1.99 (0.7–5.62)	0.195
KPS	**0.21(0.06–0.71)**	**0.012**	0.98(0.19–4.99)	0.981
Family history of cancer (Y/N)	1.43 (0.92–2.21)	0.108	0.45 (0.1–1.98)	0.289

*OR* odds ratio, *CI* confidence interval, *M* men, *W* women, *Y* yes, *N* no, the results are in bold if *p* < 0.05

### Prognosis

**Fig. 2:**
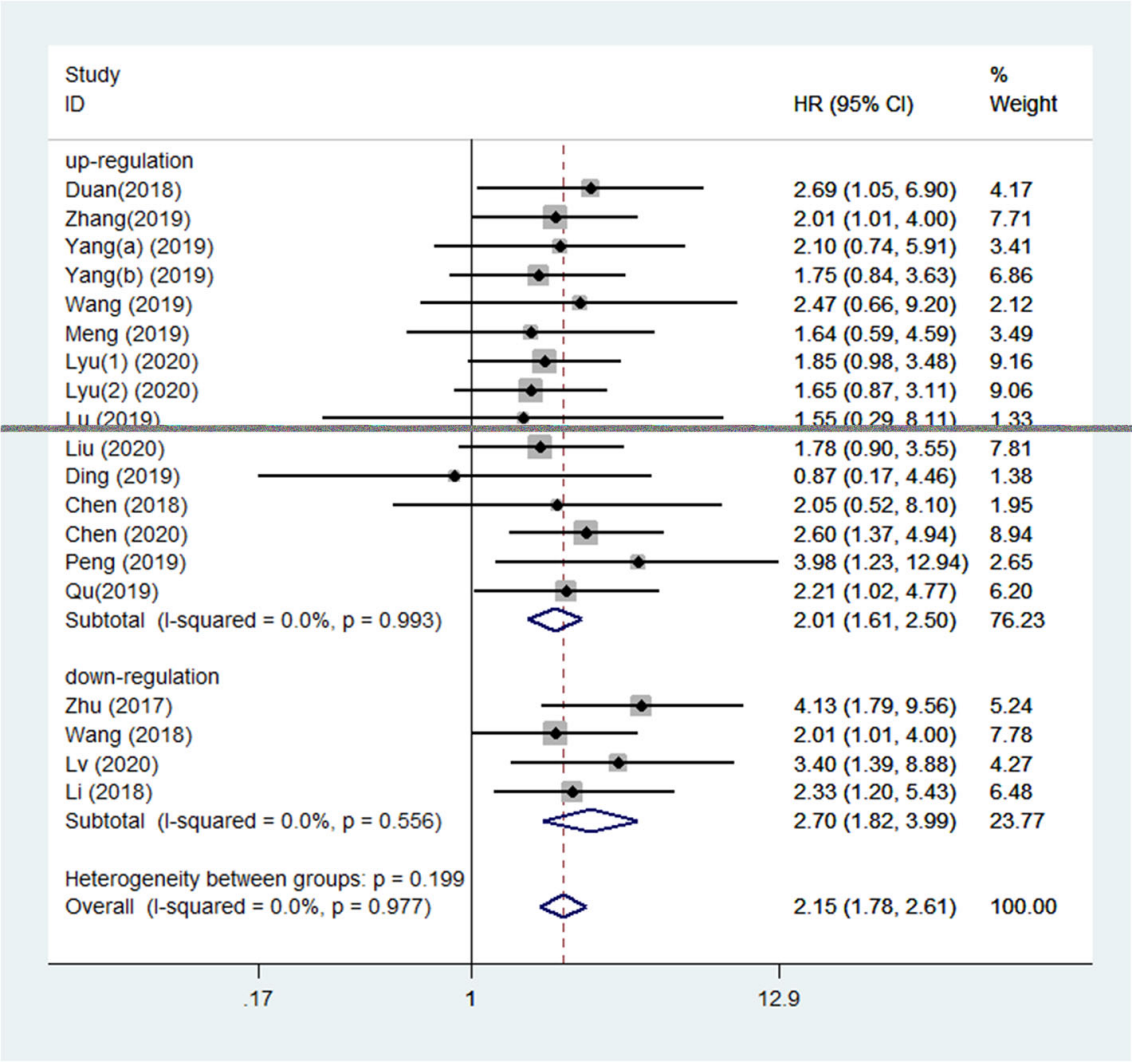
Forest plots for overall survival according to the type of upregulated circRNAs and downregulated circRNAs in glioma patients

The 18 included studies related to prognosis reported date on 19 circRNAs and 1220 glioma patients. Figure 2 shows the meta-analysis results of the relationship between circRNAs and the OS of glioma patients. The results showed that high expression of oncogenic circRNAs had a significant correlation with poor prognosis (OS: HR = 2.01, 95% CI: 1.61–2.50, *P*<0.001). The heterogeneity was not notable (*I*^2^ = 0.0%, *P* = 0.993), and we used a fixed-effect model. Moreover, high expression of tumor-suppressor circRNAs had a significant correlation with improved prognosis (OS: HR = 2.70, 95% CI: 1.82–3.99, *P*<0.001). Due to the lack of notable heterogeneity (*I*^2^ = 0.0%, *P* = 0.977), we used a fixed-effect model.

### Sensitivity analysis and publication bias

**Fig. 3:**
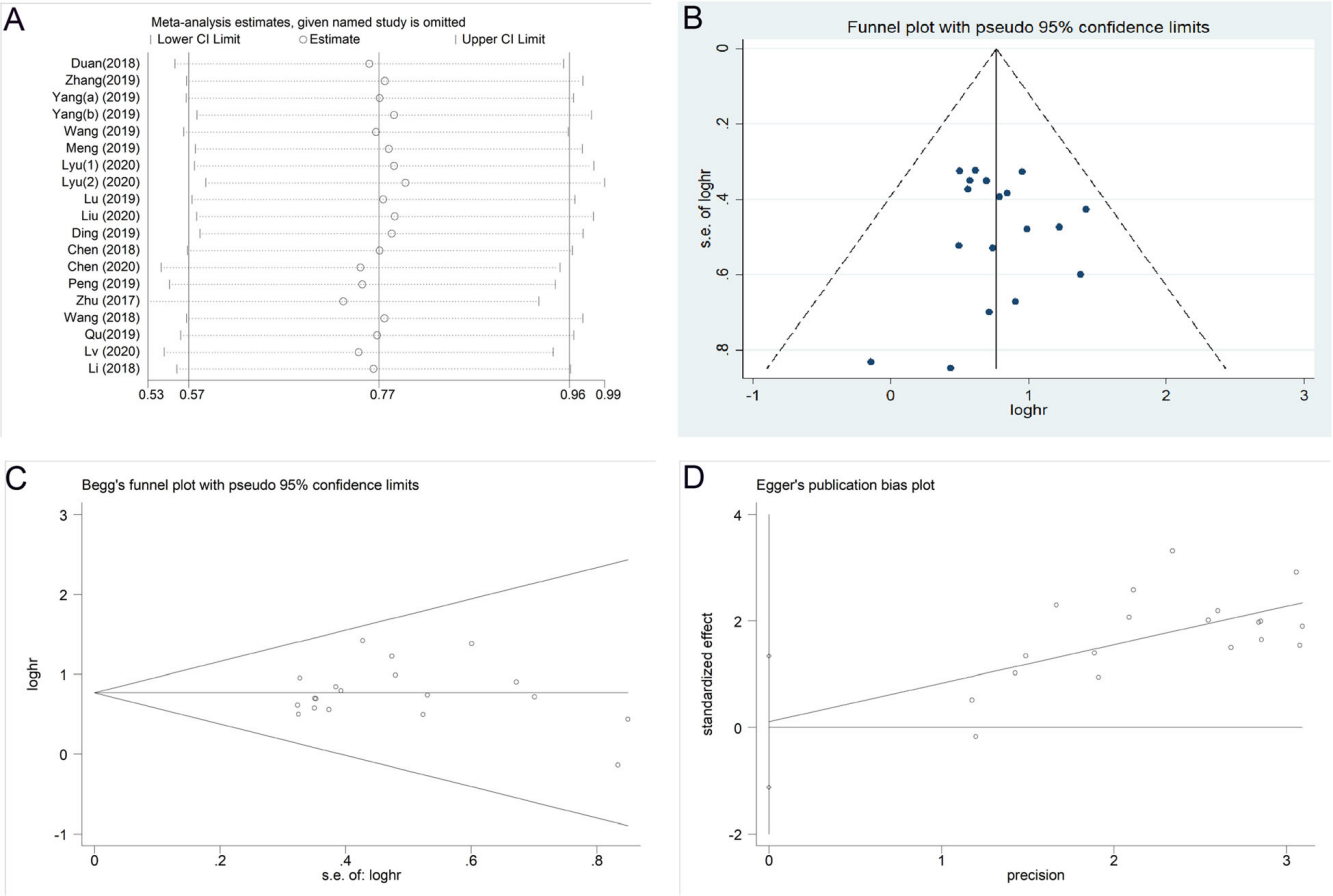
Sensitivity analysis and publication bias of the meta-analysis of circRNAs and the prognosis of patients with glioma. **a** Sensitivity analysis plot; **b** Funnel plot; **c** Begg’s funnel plot; **d** Egger’s funnel plot

The sensitivity analysis results showed that no single study had a significant effect on pooled HRs, indicating that our research results have strong reliability (Fig. 3a). As shown in Fig. 3b, we found no indication of publication bias according to the funnel chart. The result of Begg’s test is shown in Fig. 3c (*P* = 0.624), and the result of Egger’s test is shown in Fig. 3d (*P* = 0.857). Therefore, our research can exclude the possibility of publication bias.

## Discussion

With the rapid development and wide application of bioinformatics and high-throughput sequencing technology, the role of non-coding RNA has been extensively studied in various diseases. Among them, the mechanism of action of long non-coding RNA has been basically clear: in terms of epigenetic and transcriptional regulation, lncRNAs mainly complete functions by interacting with chromatin modification factors and transcription factors; in terms of post-transcriptional regulation, lncRNAs mainly act by adsorbing miRNAs to regulate miRNAs-mediated cellular processes [9]. However, the mechanism of circRNA needs further study. Studies have shown that circRNAs not only regulate the expression of downstream genes by adsorbing miRNAs, but also directly regulate transcription and interfere with the splicing process [37]. The 24 papers included in this article include 25 circRNAs, 20 of which mediate cellular processes by adsorbing miRNAs. In view of the important role of circRNA in cell growth and development, we believe that circRNA has strong application prospects like lncRNA.

Through the PubMed search, we found a large number of studies on the relationship between the expression of a single circRNA and the prognosis of glioma, but no meta- analysis of circRNA expression and the prognosis of glioma patients was found [10, 14–28, 35, 36]. This study evaluates for the first time the important relationship between circRNA expression disorders and the clinicopathological and prognostic features of glioma patients. Our study included 24 eligible studies that included a total of 1390 glioma patients. By comparing the expression levels of circRNAs with the levels in normal tissues, we divided circRNAs into two groups: carcinogenic tumor promoters and tumor suppressors.

The meta-analysis showed that the expression levels of circRNAs are significantly correlated with the clinicopathological features of glioma patients. High expression of tumor-promoter circRNAs is significantly associated with poor clinicopathological features, including age, tumor size, tumor grade, and KPS. In addition, high expression of tumor-suppressor circRNAs was significantly associated with better clinicopathological features, including gender, age, tumor size, and tumor grade. In summary, dysregulated expression of circRNAs is related to the occurrence and development of glioma, which is consistent with the current mainstream views [38].

Many single studies have reported that dysregulated expression of circRNAs is a poor prognostic factor in glioma patients based on multivariate Cox regression analysis [15–19, 35]. After a subgroup analysis, we found that circRNAs with different expression levels have different prognostic features in gliomas. Carcinogenic circRNAs are associated with a shorter OS time in glioma patients, and tumor-suppressor circRNAs are associated with a longer OS time in glioma patients. Moreover, heterogeneity testing, sensitivity analysis, and publication bias increase the reliability of the results. Therefore, circRNAs have significant clinical applications as oncogenes or tumor suppressors.

Related studies have also reported that circRNAs have great potential as novel biomarkers for glioma prognosis [38]. CircRNAs have obvious advantages as a prognostic biomarker coupled with their unique characteristics: (a) circRNAs have a covalent, closed, continuous circular structure, which is more conservative and stable than linear RNA; (b) circRNAs are widely present in eukaryotic cells and have high expression levels in cells, tissues, and body fluids; (c) compared with traditional biomarkers, circRNAs are more specific in terms of tissue expression [39, 40]. In summary, these characteristics also promote circRNA to act as valuable biomarkers for diagnosis, prognosis as well as therapeutic evaluation of glioma.

However, this study still has several limitations: (a) All eligible studies included different circRNAs, and we could not perform subgroup analysis on different circRNAs; (b) All the samples included in the study were from hospitals in China, and more studies in other regions are needed; and (c) For some studies that did not report a clear HR value, we extracted the HR value from the K-M curve, which may increase the potential for bias. Therefore, many prospective studies with larger sample sizes, more centers, and deeper exploration of functional mechanisms are needed.

## Conclusions

In conclusion, there is a significant correlation between the dysregulated expression of circRNAs and the clinicopathology and prognosis of glioma patients. Therefore, circRNAs have the potential to become novel biomarkers and therapeutic targets for the treatment of glioma.

## Supplementary information

**Additional file 1.** Table S1. Main features of studies included in the diagnosis analysis.

**Additional file 2.** Table S2. Quality assessment of eligible studies (Newcastle-Ottawa Scale).

## Data Availability

The data that support the findings of this study are available on request from the corresponding author.

## Abbreviations

AUC: Area under the curve
CI: Confidence interval
CircRNA: Circular RNA
HR: Hazard ratio
MiRNAs: MicroRNAs
NOS: Newcastle-Ottawa Scale
ORs: Odds ratios
OS: Overall survival
QRT-PCR: Quantitative real-time polymerase chain reaction
