# Supplementary material for: CircRNAs as potential biomarkers for the clinicopathology and prognosis of glioma patients: a meta-analysis
Source: BMC Cancer. 2020 Oct 15;20:1005. doi: 10.1186/s12885-020-07446-4 (PMC7566033; doi:10.1186/s12885-020-07446-4)
Supplement: Supplementary file 1 — Additional file 1. Table S1. Main features of studies included in the diagnosis analysis. [file 12885_2020_7446_MOESM1_ESM.docx]

| **Author year** | **Country** | **CircRNA** | **Tumor type** | **Expression** | **Assay methods** | **Detected sample** | **Sample size**  **Case Control** | | **SE (%)** | **SP (%)** | **AUC** | **95% CI** |
| --- | --- | --- | --- | --- | --- | --- | --- | --- | --- | --- | --- | --- |
| Li [35]2018 | China | circ-ITCH | glioma | down-regulation | qRT‐PCR | Frozen tissue | 60 | 60 | 75 | 68.33 | 0.757 | 0.670-0.830 |

**Table S1. Main features of studies included in the diagnosis analysis**

SE sensitivity, SP specificity, AUC, area under the curve, CI confidence interval, qRT-PCR quantitative reverse transcription-polymerase chain reaction
