# Supplementary material for: CircRNAs as potential biomarkers for the clinicopathology and prognosis of glioma patients: a meta-analysis
Source: BMC Cancer. 2020 Oct 15;20:1005. doi: 10.1186/s12885-020-07446-4 (PMC7566033; doi:10.1186/s12885-020-07446-4)
Supplement: Supplementary file 2 — Additional file 2. Table S2. Quality assessment of eligible studies (Newcastle-Ottawa Scale). [file 12885_2020_7446_MOESM2_ESM.docx]

**Table S2.** **Quality assessment of eligible studies (Newcastle-Ottawa Scale)**

| **Study** | **Selection** | | | **Comparability** | | | **Outcome** | | **Total** |
| --- | --- | --- | --- | --- | --- | --- | --- | --- | --- |
|  | **Adequacy of case definition** | **Number**  **of case** | **Representativeness of the cases** | **Ascertainment of**  **relevant cancers** | **Ascertainment of**  **detection method** | **CircRNA expression** | **Assessment of outcome** | **Adequate**  **follow up** |  |
| **Zhu[12]2017** | 1 | 1 | 1 | 1 | 1 | 1 | 1 | 1 | 8 |
| **Wang[13]2018** | 1 | 1 | 1 | 1 | 1 | 1 | 1 | 1 | 8 |
| **Qu[14]2019** | 1 | 1 | 1 | 1 | 1 | 1 | 1 | 1 | 8 |
| **Lv[15]2020** | 1 | 1 | 1 | 1 | 1 | 1 | 1 | 0 | 7 |
| **Duan[16]2018** | 1 | 1 | 1 | 1 | 1 | 1 | 1 | 0 | 7 |
| **Zhang[17]2019** | 1 | 1 | 1 | 1 | 1 | 1 | 1 | 1 | 8 |
| **Yang[18]2019** | 1 | 1 | 1 | 1 | 1 | 1 | 1 | 1 | 8 |
| **Yang[19]2019** | 1 | 1 | 1 | 1 | 1 | 1 | 1 | 1 | 8 |
| **Wang[20]2019** | 1 | 1 | 1 | 1 | 1 | 1 | 1 | 1 | 8 |
| **Meng[21]2019** | 1 | 1 | 1 | 1 | 1 | 1 | 1 | 1 | 8 |
| **Lyu[8]2020** | 1 | 1 | 1 | 1 | 1 | 1 | 1 | 0 | 7 |
| **Lu[22]2019** | 1 | 1 | 1 | 1 | 1 | 1 | 1 | 0 | 7 |
| **Liu[23]2020** | 1 | 1 | 1 | 1 | 1 | 1 | 1 | 1 | 8 |
| **Ding[24]2019** | 1 | 1 | 1 | 1 | 1 | 1 | 1 | 1 | 8 |
| **Chen[25]2018** | 1 | 1 | 1 | 1 | 1 | 1 | 1 | 1 | 8 |
| **Chen[26]2020** | 1 | 1 | 1 | 1 | 1 | 1 | 1 | 1 | 8 |
| **Zuo[27]2019** | 1 | 1 | 1 | 1 | 1 | 1 | 0 | 0 | 6 |
| **Zhan[28]2019** | 1 | 1 | 1 | 1 | 1 | 1 | 0 | 0 | 6 |
| **Xie[29]2018** | 1 | 1 | 1 | 1 | 1 | 1 | 0 | 0 | 6 |
| **Qian[30]2019** | 1 | 1 | 1 | 1 | 1 | 1 | 0 | 0 | 6 |
| **Liu[31]2019** | 1 | 1 | 1 | 1 | 1 | 1 | 0 | 0 | 6 |
| **He[32]2020** | 1 | 1 | 1 | 1 | 1 | 1 | 0 | 0 | 6 |
| **Peng [34]2019** | 1 | 1 | 1 | 1 | 1 | 1 | 1 | 0 | 7 |
| **Li [33]2018** | 1 | 1 | 1 | 1 | 1 | 1 | 1 | 1 | 8 |
